# Deciphering the Role of miR-200c-3p in Type 1 Diabetes (Subclinical Cardiovascular Disease) and Its Correlation with Inflammation and Vascular Health

**DOI:** 10.3390/ijms232415659

**Published:** 2022-12-10

**Authors:** Sherin Bakhashab, Megan Li Yuen Yeoh, David J. Coulson, Samuel Christian Steel, Sabina L. Ray, Jolanta U. Weaver

**Affiliations:** 1 Biochemistry Department, King Abdulaziz University, Jeddah 21589, Saudi Arabia; 2 Translational & Clinical Research Institute, Newcastle University, Newcastle upon Tyne NE2 4HH, UK; 3 Department of Diabetes, Queen Elizabeth Hospital, Gateshead, Newcastle Upon Tyne NE9 6SH, UK; 4 Vascular Biology and Medicine Theme, Newcastle University, Newcastle Upon Tyne NE2 4HH, UK

**Keywords:** cardiovascular disease, circulating endothelial progenitor cells, miR-200c-3p, inflammation, proangiogenic cells, type 1 diabetes mellitus

## Abstract

Uncomplicated type 1 diabetes (T1DM) displays all features of subclinical cardiovascular disease (CVD) as is associated with inflammation, endothelial dysfunction and low endothelial progenitor cells. MiR-200c-3p has been shown in animal tissues to be pro-atherogenic. We aimed to explore the role of miR-200c-3p in T1DM, a model of subclinical CVD. 19 samples from T1DM patients and 20 from matched controls (HC) were analyzed. MiR-200c in plasma and peripheral blood mononuclear cells (PBMCs) was measured by real-time quantitative polymerase chain reaction. The results were compared with the following indices of vascular health: circulating endothelial progenitor cells, (CD45^dim^CD34^+^VEGFR-2^+^ or CD45^dim^CD34^+^CD133^+^) and proangiogenic cells (PACs). MiR-200c-3p was significantly downregulated in PBMCs but not in plasma in T1DM. There was a significant negative correlation between the expression of miR-200c-3p and HbA1c, interleukin-7 (IL-7), vascular endothelial growth factor-C (VEGF-C), and soluble vascular cell adhesion molecule-1, and a positive correlation with CD45^dim^CD34^+^VEGFR-2^+^, CD45^dim^CD34^+^CD133^+^ and PACs. Receiver operating curve analyses showed miR-200c-3p as a biomarker for T1DM with significant downregulation of miR-200c-3p, possibly defining subclinical CVD at HbA1c > 44.8 mmol/mol (6.2%). In conclusion, downregulated miR-200c-3p in T1DM correlated with diabetic control, VEGF signaling, inflammation, vascular health and targeting VEGF signaling, and may define subclinical CVD. Further prospective studies are necessary to validate our findings in a larger group of patients.

## 1. Introduction

Cardiovascular disease (CVD) contributes greatly towards premature deaths, especially in type 1 diabetes mellitus patients (T1DM) [1,2,3]. Ischemic heart disease, for instance, causes 36% of life expectancy losses in males and 31% in females, of those with T1DM [4]. Unfortunately, the mechanism behind the increased risk is still not clearly understood [1].

According to the Diabetes Control and Complications Trial conducted in 2005, intensive treatment, together with ensuring that T1DM patients’ glycosylated hemoglobin (HbA1c) was <6.5%, could reduce their risk of CVD by 42%. However, achieving such a target is not an easy feat and the majority of T1DM patients are unsuccessful. Therefore, there is a need to explore an alternative way of reducing their risks of CVD to improve their life expectancy and quality.

Several of our studies have showed that early features of CVD are common in T1DM patients, despite them never having had any CVD events [5,6,7]. For instance, T1DM patients without history of CVD have reduced circulating endothelial progenitor cells (cEPCs) [5,6]. It is well established that cEPCs contribute to endothelial repair, and having a reduced number increases the risk of cardiovascular events and deaths [8]. Additionally, T1DM patients are characterized by the presence of inflammation, even in very fit and young individuals, which also contributes to endothelial dysfunction [7,9]. The increased arterial stiffness in T1DM patients versus healthy subjects is seen as early as the age of 10 to 18 [10,11]. Other studies have also found that 9 to 22-year-old T1DM patients have increased carotid intima-media thickness synonymous with subclinical CVD [12,13,14,15]. Since endothelial dysfunction, increased arterial stiffness and carotid intima-media thickness in T1DM patients are precursors for established CVD, T1DM is thus widely recognized as subclinical CVD [16,17,18,19,20]. On the basis of premature CVD in T1DM in the UK, the National Institute for Clinical Excellence (NICE) recommends that T1DM patients should be routinely offered statin therapy if they are above the age of 40, or have had the disease for at least 10 years [21]. Despite all the efforts to reduce CVD risk in T1DM, the outcome of managing CVD in diabetes is worse than in nondiabetic patients [22,23]. Therefore, there is a clinical need to explore the therapeutic opportunities for future management of prevention/attenuation of CVD, particularly in T1DM. 

It is well established that microRNAs (miRNAs) play crucial regulatory roles in the pathogenesis of CVD. MiRNAs also play important roles in immune responses as well as inflammation. They are small RNAs with 22 non-coding nucleotides. MiRNAs bind to 3′ untranslated regions of mRNAs, and either block translation or promote mRNA degradation, disrupting protein synthesis. Their expression is tissue and disease specific. Furthermore, as miRNAs are easily detected in PBMCs and plasma, they are ideal potential therapeutic targets or biomarkers [24].

Animal studies have found evidence on miR-200c, a miRNA isoform, in vascular smooth muscle cells (VSMCs), aortic endothelial cells and cardiomyocytes of diabetic animals or patients, that is consistent with the pro-atherogenic/inflammatory effects of miR-200c in the studied tissues [25,26,27]. Specifically, upregulation of miR-200c was found in VSMCs or aortas in type 2 diabetic mice (db/db mice), whilst miR-200c target, Zeb1, an E-box binding transcriptional repressor was downregulated [25]. Furthermore, the transfection of miR-200 mimics into VSMCs in control mice, downregulated Zeb1, upregulated the inflammatory genes cyclooxygenase-2 (COX-2) and monocyte chemoattractant protein-1, and promoted monocyte binding to VSMCs. This effect was reversed by using miR-200c inhibitors [25]. 

Similarly, an increased expression of miR-200c was observed in a model of diabetic cardiomyopathy in Streptozocin rats and in cardiomyocytes exposed to high glucose levels [26]. The subsequent reversal of cardiomyocytes’ hypertrophy was achieved by inhibition of miR-200c [26]. Moreover, an upregulated expression of miR-200c was found in arteries from diabetic mice and renal arteries from patients with diabetes, compared with arteries from non-diabetic mice or human subjects [27]. Furthermore, an overexpression of miR-200c in non-diabetic endothelial cells (EC) lead to reduced endothelium-dependent relaxation (EDR), and reversal was achieved by use of anti-miR-200c in diabetic mice. It was postulated that miR-200c may be a useful target for interventions in vascular diseases [27].

Since animal research has proven the mechanisms involved in target tissues elaborate inflammation and endothelial dysfunction, it is paramount to establish the role of circulating miR-200c in plasma or in peripheral blood mononuclear cells (PBMCs) in diabetic patients in vascular repair. We thus hypothesize that miR-200c is increased in plasma or PBMCs in a state of increased inflammation such as subclinical CVD. We therefore studied samples from healthy control (HC), and T1DM patients without known CVD but with features of subclinical CVD.

## 2. Results

### 2.1. Subjects’ Characteristics

The clinical and metabolic characteristics of the original cohort of subjects were previously represented [5]. The T1DM subjects have had T1DM for 22.4 ± 13.9 years and their blood glucose levels were relatively well controlled (HbA1c 57.3 ± 7.6 mmol/mol), with no history of CVD. The other features of subclinical CVD are supported by the reduction of cEPCs, Hills colonies PACs, fibronectin adhesion assay (FAA) and an increase in cECs [5]. 

### 2.2. Cytokine Profiles

We have previously reported that T1DM patients studied by us showed features of chronic inflammation. Patients had significantly elevated homeostatic cytokine, interleukin-7 (IL-7, 2.3 ± 0.6 pg/mL), pro-inflammatory cytokines IL-8 (4.7 ± 1.3 pg/mL) and tumour necrosis factor-alpha (TNF-α, 1.6 ± 0.2 pg/mL), as well as growth factor vascular endothelial growth factor-C (VEGF-C, 63.2 ± 20.3 pg/mL) when compared with HCs (1.4 ± 0.6 pg/mL, 2.8 ± 0.5 pg/mL, 1.4 ± 0.2pg/mL and 50.8 ± 48.2 pg/mL, respectively); *p* = 0.008, *p* = 0.003, *p* = 0.041 and *p* = 0.013, respectively as reported by us [28]. The mRNA expressions of CXCR1 and CXCR2 were 4.3 (*p* = 0.009) and 2.3 (*p* < 0.001) fold-change (FC) higher in T1DM patients, respectively, which was previously documented by us [28].

### 2.3. Expression of hsa-miR-200c-3p 

**Figure 1:**
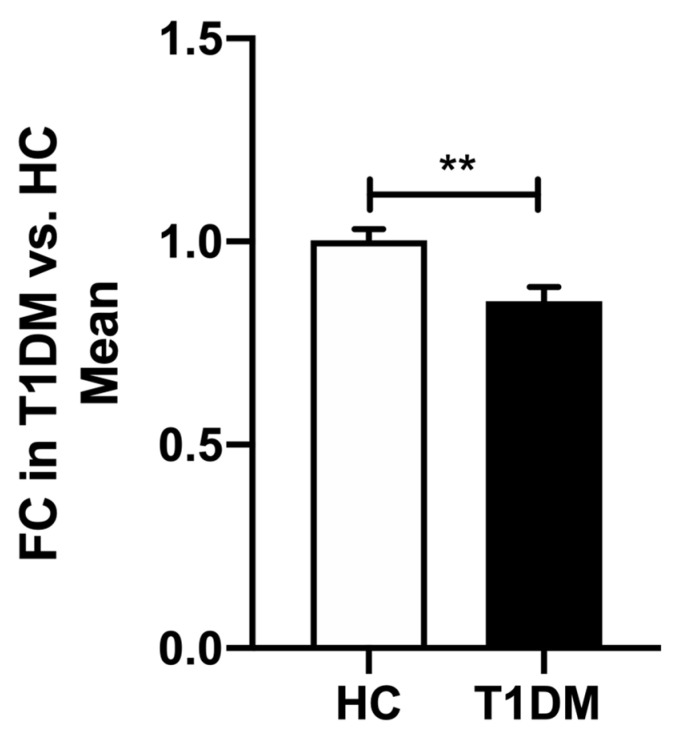
Comparison of miR-200c-3p expression between HC and T1DM patients in PBMCs. Data are represented as mean ± SEM and analyzed by unpaired *t*-tests. ** *p* < 0.01.

miR-200c-3p was significantly downregulated in PBMCs of T1DM patients when compared with HCs, FC = −1.2, *p* = 0.006 (Figure 1); but not in plasma FC = −0.662, *p* = 0.378. 

### 2.4. Correlation between HbA1c and hsa-miR-200c-3p

**Figure 2:**
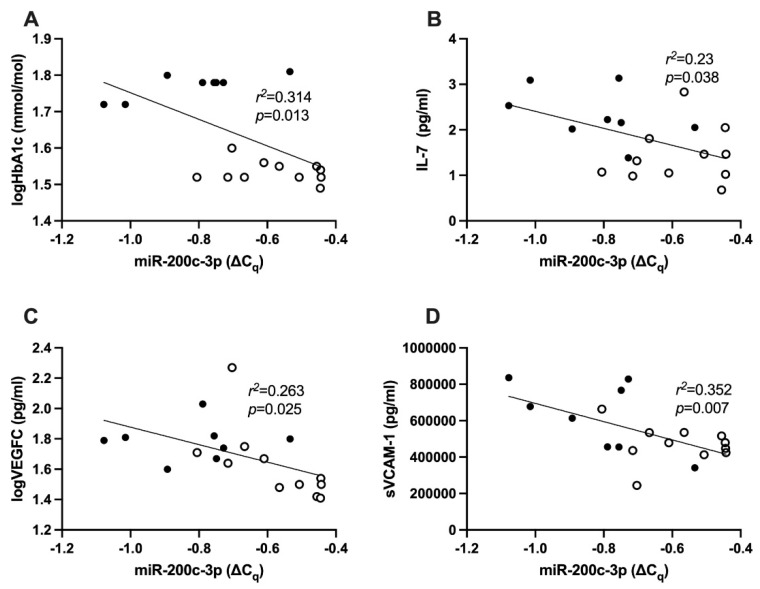
(**A**) Correlation between miR-200c-3p in PBMCs and HbA1c (r^2^ = 0.314, *p* = 0.013) as a parameter to portray the glycemic control across study groups, (**B**) correlation between miR-200c-3p in PMBC and IL7 (r^2^ = 0.23 *p* = 0.038), (**C**) logVEGFC (r^2^ = 0.263, *p* = 0.025), (**D**) sVCAM-1 (r^2^ = 0.352, *p* = 0.007), across study groups in the plasma. Statistical analysis was carried out using linear regression analysis. IL: interleukin; VEGFC: vascular endothelial growth factor C; sVCAM-1: circulating vascular cell adhesion molecule 1.

There was a significant negative correlation between all subjects’ log HbA1c and the expression of miR-200c-3p, r^2^ = 0.314, *p* = 0.013 (Figure 2A). The residual plot for the correlation between log HbA1c and miR-200c-3p was calculated (Supplementary Material Figure S1A).

### 2.5. Correlation between hsa-miR-200c-3p and Cytokines

There was a significant negative correlation between miR-200c-3p expression with the expression of IL-7 (r^2^ = 0.23, *p* = 0.038, Figure 2B), log VEGF-C (r^2^ = 0.263, *p* = 0.025, Figure 2C), and soluble vascular cell adhesion molecule 1 (sVCAM-1, r^2^ = 0.352, *p* = 0.007, Figure 2D) in all subjects. The residual plots for the correlation between miR-200c-3p and IL-7, log VEGF-C and sVCAM-1 have been documented (Supplementary Material Figure S1B–D).

### 2.6. Correlation between miR-200c-3p and cEPCs

**Figure 3:**
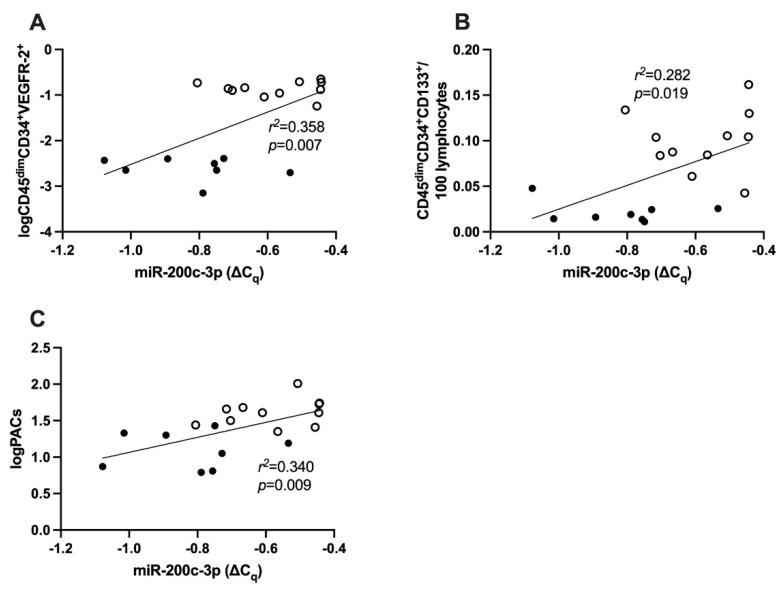
Correlation between miR-200c-3p expression in PBMCs and vascular health in type 1 diabetes and healthy controls combined. (**A**) miR-200c-3p and CD45^dim^CD34^+^VEGFR-2^+^ (**B**) miR-200c-3p and CD45^dim^CD34^+^CD133^+^; (**C**) miR-200c-3p and logPACs. Statistical analysis was carried out using linear regression analysis. CD: cluster of differentiation; PAC: proangiogenic cells.

MiR-200c-3p expression was significantly positively correlated with log (CD45^dim^CD34^+^VEGFR-2^+^ per 100 lymphocytes) (r^2^ = 0.358, *p* = 0.007, Figure 3A) as well as CD45^dim^CD34^+^CD133^+^ per 100 lymphocytes (r^2^ = 0.282, *p* = 0.019, Figure 3B) in all subjects. The residual plots for the correlation between miR-200c-3p and log (CD45^dim^CD34^+^VEGFR-2^+^ per 100 lymphocytes), and CD45^dim^CD34^+^CD133^+^ per 100 lymphocytes were calculated (Supplementary Material Figure S2A,B). Optimization of the gating strategy for flow cytometric analysis of cEPCs is explained in Supplementary Material Figure S4.

### 2.7. Correlation between miR-200c-3p and PACs

In all subjects, miR-200c-3p was also significantly positively correlated with log PACs (r^2^ = 0.340, *p* = 0.009, Figure 3C). The residual plot for the correlation between log PACs and miR-200c-3p is shown in Supplementary Material Figure S3. Enumeration of PAC using Ulex lectin and DiLDL (1,19–dioctadecyl–3,3,39,39–tetramethylindocarbocyanine–labeled acetylated low-density lipoprotein) was added to the Supplementary Material and representative staining images (Figure S5). 

### 2.8. Receiver Operating Characteristic Curve Analysis of miR-200c-3p and T1DM

**Figure 4:**
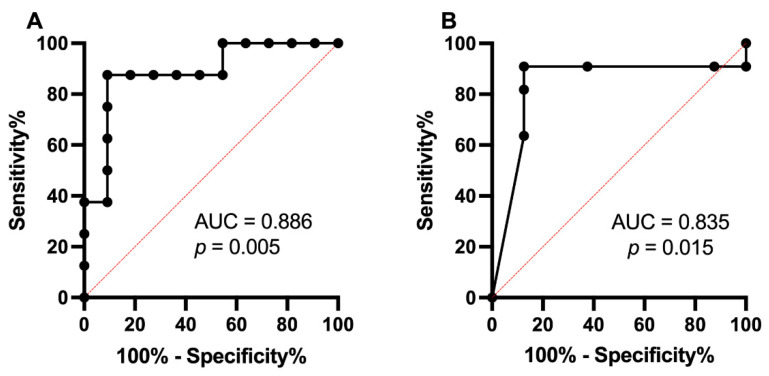
(**A**) Receiver Operating Characteristic Curve (ROC) for miR-200c-3p in type 1 diabetes and healthy controls; (**B**) ROC curve of HbA1c indicating downregulation of miR-200c-3p expression.

Receiver operating characteristic curve (ROC) analyses showed that miR-200c-3p was able to distinguish between T1DM and HC (AUC = 0.886, *p* = 0.005) with a sensitivity of 87.5% and 90.91% specificity (Figure 4A). In addition, significant downregulation of miR-200c-3p (AUC = 0.835, *p* = 0.015) with a sensitivity of 90.91% and 87.5% specificity defined subclinical CVD at HbA1c > 44.8 mmol/mol (6.2%), *p* = 0.015 (Figure 4B).

## 3. Discussion

### 3.1. Inflammation in T1DM

This study has confirmed that T1DM patients without known CVD had inflammation, as plasma levels of pro-inflammatory cytokines IL-8 and TNF-α were elevated, as demonstrated by us earlier and in previous studies [7,29]. Additionally, the homeostatic cytokine IL-7 was elevated. This cytokine was found to contribute to inflammation, because according to a recent mechanistic study, upon downregulating IL-7 in the intra-epithelial lymphocytes of mice with colitis, inflammation could be reduced [30]. Furthermore, another cytokine VEGF-C, which increases inflammation, was elevated in T1DM patients. According to a gene therapy study, upon increasing VEGF levels in mice cerebra via recombinant adeno-associated virus, unwanted inflammation was promoted [31]. 

### 3.2. miR-200c-3p Expression in T1DM

Our finding of reduced miR-200c in PBMCs in T1DM is concordant with previously published research [32]. In another inflammatory state, chronic psoriasis, which can be considered subclinical CVD, miR-200c was equally downregulated in PBMCs [33]. Furthermore, others have documented in the study on cord blood that downregulation of miR-200c was within CD34+ hematopoietic progenitor cells, versus other lineage cells: T cells, granulocyte cells and mononuclear cells [34]. Although our hypothesis has been rejected, our results are consistent with the role of miR-200c in hematopoiesis and vascular health. Based on previous studies involving murine models, miR-200c was found to be upregulated in target cells/organs for vascular health: endothelial cells, cardiomyocytes, and VSMCs in diabetic models (animals or cells) [25,26,27]. This confirms not only previously described tissue specific expression of miRNAs, but also that miR-200c is invariably upregulated in tissue affected by inflammation (target cells) [24,35]. 

### 3.3. miR-200c-3p and Inflammation

We found a negative correlation between miR-200c-3p in PBMCs and IL-7, VEGF-C and sVCAM. Therefore, the higher the number of circulating cytokines, the lesser miR-200c in PBMCs in our subjects. Thus, cytokines released by PBMCs may have an effect on miR-200c expression in PBMCs, suggesting a negative feedback loop between miRs and cytokines, thus possibly promoting greater inflammatory response. Soluble VCAM-1 has been found to contribute to the attachment of inflammatory cells to the vascular endothelial wall, as well as the promotion of their subsequent migration through the endothelium, accelerating atherosclerosis [36,37]. In an animal model of atherosclerosis, miRNA-200c-3p overexpression in the angiotensin II-induced rat renal artery EC markedly inhibited cell proliferation and migration [38]. The inverse correlation between miR-200c and VEGF signaling suggests that the greater the expression of miR-200c, the lower the number of VEGF-C cytokines involved in angiogenesis. Thus, this supports the anti-angiogenic effect of miR-200c. In patients with IgA nephropathy, the level of intrarenal miR-200c was also downregulated and associated with proteinuria [39]. The tissue specific expression of miR-200c should not be seen in isolation. Diverse expression of miR-200c has also been noted in different cancer cells, suggesting that various factors may be involved in miR-200c tissue expression. 

In diabetic animals, miR-200c iR-200c was upregulated in mice or rat diabetic hearts [40,41]. In addition, according to a study conducted by Zhang et al. in 2016, miR-200c contributed to inflammation via suppression of Zeb1, causing COX-2 upregulation [27]. This in turn caused the impairment of endothelium-dependent relaxations in mouse aortas [27]. Upon administration of anti-miR-200c, the elevation of COX-2 expression was reversed [27]. Additionally, in 2012, a study by Reddy et al. demonstrated that transfection of miR-200 family members into VSMCs downregulated Zeb1 and consequently upregulated COX-2 [25]. Additionally, a study by Saito et al. in 2016 found that reactive oxygen species were increased by the overexpression of miR-200c in rats’ cardiomyocytes via decreased mitochondrial superoxide dismutase and catalase activities, enhancing ischemia or reperfusion injury [41]. Knockdown of this miRNA decreased the reactive oxygen species levels significantly [41]. These three studies showed that miR-200c was positively associated with inflammation. These results are not necessarily contradictory to our results as different tissues were studied; i.e., PBMCs involved in vascular repair, versus target organs such as endothelial or smooth muscle cells. The best example to illustrate this point comes from research in patients with a chronic inflammatory state (severe psoriasis), where miR-200c was upregulated in the inflamed skin but downregulated in PBMCs in the same disease group [33,35]. 

**Figure 5:**
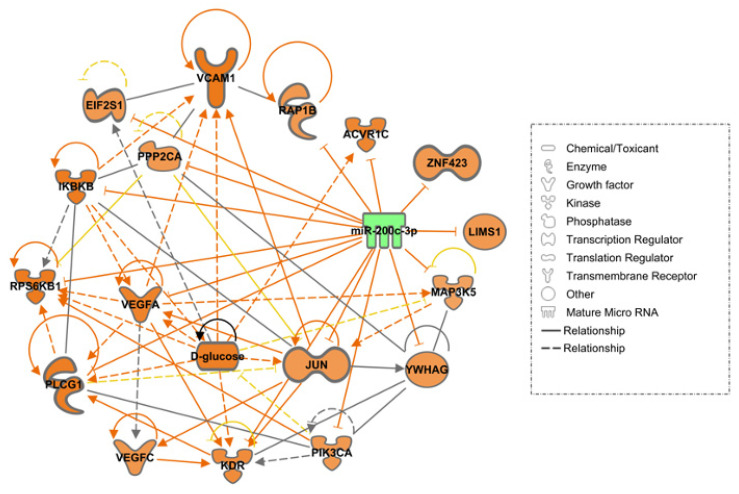
Ingenuity Pathway Analysis (IPA) prediction network of miR-200c-3p using our data and its mRNA targets, supporting its involvement in cardiovascular disease. Three pathways were considered relevant in this study: the PI3K-Akt signaling pathway, the Hippo signaling pathway, and the VEGF signaling pathway. The green color denoted decreased measurement, and the orange color denoted upregulation or predicted activation. The lines colored in orange represented stimulation, blue inhibition and grey effect not predicted. Solid lines represented a direct relationship, whereas dashed, interrupted ones represented an indirect relationship. Genes outlined with blue are involved in VEGF signaling, while ones outlined in dim gray are involved in PI3K/Akt signaling pathways. EIF2S1: Eukaryotic Translation Initiation Factor 2 Subunit α; IKBKβ: Inhibitor of Nuclear Factor Kappa B Kinase Subunit β; JUN: Jun Proto-Oncogene, AP-1 Transcription Factor Subunit; KDR: kinase insert domain receptor (VEGFR-2); MAP3K5: Mitogen-Activated Protein Kinase Kinase Kinase 5; PIK3CA: Phosphatidylinositol-4,5-Bisphosphate 3-Kinase Catalytic Subunit α; PLCG1: Phospholipase C Gamma 1; PPP2CA: Protein Phosphatase 2 Catalytic Subunit Alpha; RAP1B: RAP1B, Member Of RAS Oncogene Family; RPS6KB1: Ribosomal Protein S6 Kinase B1; VCAM1: Vascular Cell Adhesion Molecule 1; VEGFA: vascular endothelial growth factor A; VEGFC: vascular endothelial growth factor C; YWHAG: Tyrosine 3-Monooxygenase/Tryptophan 5-Monooxygenase.

In our study, by inputting hsa-miR-200c-3p, IL-7, VCAM-1 and VEGF-C into the IPA analysis together with glucose to simulate the diabetic state, we predicted that downregulated miR-200c may lead to cardiovascular disease through VEGF signaling and PI3K/Akt signaling pathways (Figure 5). The prediction did not show whether there was direct or indirect interaction between miR-200c-3p and IL-7.

We have shown for the first time that miR-200c in PBMCs was inversely correlated with the prevailing glycemic level across the study, both in controls and patients with T1DM. It is well established that a degree of hyperglycemia is causal to CVD. Indeed, hyperglycemia is the principal cause of induced oxidative stress; therefore, exposing ECs to high glucose led not only to oxidative stress but also to upregulation in miR-200c, which subsequently induced EC apoptosis and senescence via Notch1 inhibition [42,43]. Since hyperglycemia augments miR-200c expression in target cells (EC), but not in the immune regulatory cells such as PBMCs, a possible explanation is that miR-200c expression may be downregulated indirectly by factors such as proinflammatory cytokines; as detected by previous studies, IL-6 is negatively correlated with miR-200c-3p expression in inflammatory diseases [44,45] and also IL-8 [46]. 

### 3.4. miR-200c-3p and Vascular Health

The cEPCs are bone marrow-derived precursors to ECs that promote vascular repair in response to vascular damage [47]. A pivotal study has shown that increased cEPCs are associated with reduced risk of CVD-related deaths [8]. According to our study, T1DM patients with good diabetic control had reduced cEPCs when compared with HC [5]. 

In the current study we have shown hsa-miR-200c-3p in PBMCs to be positively correlated with cEPCs (logCD45^dim^CD34^+^VEGFR-2^+^ per 100 lymphocytes and CD45^dim^CD34^+^CD133^+^ per 100 lymphocytes). CD45^dim^CD34^+^VEGFR-2^+^ referred to matured cEPCs, whereas CD45^dim^CD34^+^CD133^+^ referred to immature cEPCs. Both were associated with reduced risk of CVD-related deaths. 

Recent evidence has shown that miR-200c can inversely modulate myeloid differentiation factor 88 (Myd88) [48]. Furthermore, attenuating Myd88 signaling can alleviate inflammation and fibrosis in fetal-derived human placental mesenchymal stem cells [49]. Similarly, the effect of miR-200c has also been studied on bone marrow and human bone mesenchymal stem cells, showing that overexpression of miR-200c enhances proliferation of osteoblasts [50]. 

Another indicator of vascular function (cell adhesion) studied by us is defined by PACs, derived from PBMCs previously known as early endothelial progenitor cells. In T1DM, poor glycemic control or a hyperglycemic condition were associated with the reduction of PACs, contributing to increased risk of CVD [51,52,53]. Our study has shown that miR-200c-3p was also positively correlated with PACs. Since the reduction of PACs could lead to increased risks of CVD, as mentioned previously, the downregulation of hsa-miR-200c-3p in PBMCs by hyperglycemia in T1DM patients, leading to the reduction of PACs, would also be one of the factors which could increase their risk of CVD [51,52,53].

ROC analysis showed that miR-200c-3p expression was able to distinguish between subclinical CVD (T1DM) and controls with high sensitivity and specificity. The significant downregulation of miR-200c-3p defined subclinical CVD at HbA1c > 44.8 mmol/mol (6.2%). Thus, miR-200c can be considered a biomarker for subclinical CVD, as the difference in its expression is related to rising glycemic levels. The value of HbA1c of 44.8 mmol/mol or 6.2% is considered close to the threshold of HbA1c 6.4% for the development of vascular complications in diabetes, published by Detect-2 group [54]. Thus, the onset of subclinical CVD occurs within the pre-diabetes range of 42–47 mmol/mol (IFCC) or 6.0–6.4% (DCCT). Our finding has a highly relevant clinical significance, as it has the potential to replace invasive methods of defining subclinical CVD with miR biomarkers for diagnostic and prognostic applications.

### 3.5. Causation/Contribution

mir-200c-3p has an effect on 24 pathways (mirPath v3), many of which are cross related. Since our clinical study relies on comparisons and correlations, the contribution or causality may be drawn from miR-200c target genes’ binding sites, as listed in Table 1. MiR-200c-3p has binding sites in 12 target genes of interest. The highest number of genes with miR-200c-3p binding sites was found among the pathways of interest: the VEGF signaling pathway and PI3K-Akt signaling pathway.

## 4. Materials and Methods

### 4.1. Study

In this cross-sectional study, T1DM patients with good glycemic control (HbA1c 7.4 ± 0.7% [57.3 ± 7.6 mmol/mmol]) and free from diabetic complications (active proliferative retinopathy, stage 3b renal impairment, i.e., eGFR < 45mL/min/1.73m^2^) or macrovascular disease were recruited. There were 29 T1DM patients and 20 HCs, matched for age and sex. Blood samples were obtained after an overnight fast. 

This study was carried out in line with the Helsinki Declaration. T1DM subjects were recruited from either Queen Elizabeth Hospital, Gateshead or Royal Victoria Infirmary, Newcastle, United Kingdom. Approval from the NHS Health Research Authority, NRES Committee Northeast-Sunderland, United Kingdom (Research Ethics Committee Reference Number 12/NE/0044) as well as informed consent from all subjects were obtained.

### 4.2. Cytokine Analysis

K15050D V-PLEX Cytokine Panel 1 human kit, K15049D V-PLEX Pro-inflammatory Panel 1 human kit, K15190D V-PLEX Angiogenesis Panel 1 human kit and K151JFC human TIMP-1 kit (Meso Scale Discovery, Rockville, MD, USA) were used to assay IL-7, IL-8, VEGF-C, TNF-α and sVCAM-1 in plasma samples from patients and HCs, according to the manufacturer’s protocol. Meso Scale Discovery Sector Imager 2400 was used to read plates, while Meso Scale Discovery Workbench 2.0 software was used to analyze the data.

### 4.3. Plasma miRNA Extraction

Blood samples were centrifuged at 500× *g* for 15 min. The platelet-rich plasma (upper fraction of the centrifuge) was then re-centrifuged at 13,000× *g* for another 5 min to obtain platelet-free plasma, which then was stored at −80 °C to be analyzed later. Hemolysis was checked for all samples. To monitor hemolysis, two microRNAs are used: one that is expressed in red blood cells (miRNA-451), and one that is relatively stable in serum and plasma and not affected by hemolysis (miRNA-23a). Samples with ratios above 7.0 have an increased risk of being affected by hemolysis [55]. 

An aliquot of 200 μL per sample was transferred to a FluidX tube and 60 μL of lysis solution BF, containing 1μg carrier-RNA per 60 μL lysis solution BF and RNA spike-in template mixture, was added to the sample and mixed for 1 min and incubated for 7 min at room temperature. This was followed by the addition of 20 μL protein precipitation solution BF. The total RNA was extracted from the samples using miRCURY RNA isolation kit-Biofluids and high-throughput bead-based protocol (Exiqon, Vedbaek, Denmark) in an automated 96-well format. The purified total RNA was eluted in a final volume of 50 μL. Plasma miRNAs were isolated from plasma samples using RNA isolation protocol, optimized for plasma by QIAGEN (Exiqon Services, Denmark). Agilent 2100 (Santa Clara, CA, USA) was used to assess the RNA samples’ integrity, giving high RNA integrity numbers (RIN) that ranged from 9.1 to 10.

### 4.4. Peripheral Blood Mononuclear Cell Total RNA Extraction

Ficoll (GE Healthcare, Chicago, IL, USA) separation was used to isolate PBMCs from peripheral blood. A Trizol lysis buffer was used to lyse cells, and the lysates were stored at −80 °C for later analysis. An miRNEasy Kit (QIAGEN, Hilden, Germany) was used to extract total RNA from PBMCs. Agilent 2100 (Santa Clara, CA, USA) was used to assess the integrity of RNA samples yielding high RIN ranging between 9.1 and 10.

### 4.5. Quantitative Real-Time Polymerase Chain Reaction for miRNA

The miRCURY LNA RT Kit (QIAGEN, Hilden, Germany) was used to assay miRNA quantitative real-time polymerase chain reaction (qRT-PCR) in plasma and PBMCs as described previously in detail [56]. Briefly, cDNA, from the reverse transcription of 10ng RNA extracted from plasma or PBMCs, was diluted 100× and assayed according to miRCURY LNA miRNA PCR protocol in 10 μL PCR reactions. MiRNA Ready-to-Use PCR, Pick and Mix and miRCURY LNA SYBR Green master mix were used to assay miRNA by quantitative PCR. Negative control was also obtained from the reverse transcription reaction and profiled comparably to the samples. The LightCycler^®^ 480 Real-Time PCR System (Roche, Basel, Switzerland) was used to perform amplification in 384-well plates. Roche LC software was used to analyze the amplification curves to obtain ∆Ct values. Hsa-miR-200c-3p was assayed using miRCURY LNA miRNA PCR Assays with Cat number: YP00204482 (Qiagen). Normalization was performed based on the average of the assays detected in all samples, using global mean normalization method; the assays used are listed in the Supplementary Material. Fold-change analysis was performed using 2^−ΔΔCq^ calculation.

### 4.6. In Vitro Assays for Vascular Health

#### 4.6.1. Evaluation of Circulating Endothelial Progenitor Cells (cEPCs) and Circulating Endothelial Cells (cECs) via Flow Cytometry

cEPCs were defined as CD45^dim^CD34^+^VEGFR-2^+^ cells and cECs as CD45^dim^CD133^−^ CD34^+^; events were measured by flow cytometry on a BD FACS CantoTM II system (BD bioscience, San Jose, CA, USA) as described previously [5].

#### 4.6.2. Proangiogenic Cells (PACs) In Vitro Assay

Enumeration of proangiogenic cells (PACs) in vitro assay was previously described by us [5].

### 4.7. Ingenuity Pathway Analysis (IPA)

The functional pathways, cellular functions, and target genes regulated by miR-200c-3p were identified using IPA software version 9.0 (Ingenuity, Redwood City, CA, USA).

The interaction site prediction between the transcripts and miR-200c-3p was performed using TargetScan Human, release 7.1 (www.targetscan.org, accessed on 3 June 2021) and Diana-TarBase v8, (http://carolina.imis.athena-innovation.gr/diana_tools/web/index.php?r=tarbasev8%2Findex, accessed on 3 June 2021) [57] databases.

### 4.8. Statistical Analyses

All data was presented as mean ± standard deviation, unless otherwise stated. The Shapiro–Wilk test was used to assess the normality of the data. For those that had *p* < 0.05, the data was log transformed before further analyses. The significance of the data was assessed using unpaired *t*-tests. Correlation between miR-200c-3p expression and other markers was measured using linear regression tests. Receiver operating characteristic (ROC) curve analysis of miR-200c-3p in study participants was carried out to assess the sensitivity of miR-200c-3p as a biomarker for subclinical CVD/T1DM. Moreover, ROC curve analysis of significantly decreased miR-200c and HbA1c was performed to determine the cut-off value for miR-200-3p downregulation. Statistical analysis was performed using GraphPad Prism 9.0 (GraphPad software, San Diego, CA, USA). A *p* < 0.05 was considered statistically significant.

## 5. Conclusions

MiR-200c-3p is downregulated in PBMCs in subclinical CVD (T1DM), and may define early CVD. Its direct gene targets in VEGF signaling potentially contribute to reduced angiogenesis. Its correlation with indices of vascular health confirms its role in the vascular biology of CVD. Further prospective studies are necessary to validate our findings in a larger group of patients.

## Figures and Tables

**Table 1:** Predicted consequential pairing of target regions in the transcript and miR-200c-3p.

Target Gene	Representative Transcript	Gene Name	Transcript Position	Predicted Consequential Pairing of Target Region in Transcript (Top) and miRNA (Bottom)	Site Type
EIF2S1	ENSG00000134001	Eukaryotic Translation initiation factor 2 Subunit α	58–85 3′UTR	(Transcript) 5′AUCCUAGACUUGAAAGUUUUCCAGUAUUG3′ (miRNA) 3′AGGUAGUAAUGGGCCGUCAUAAU5′	7mer
IKBKB	ENSG00000104365	Inhibitor of Nuclear Factor Kappa B Kinase Subunit β	2719–2739 3′UTR	(Transcript) 5′CCUGUCUCUCACAGCAUCUACAGUAUUA3′ (miRNA) 3′AGGUAGUAAUGGGCCGUCAUAAU5′	9mer
JUN	ENSG00000177606	Jun Proto-Oncogene, AP-1 Transcription Factor Subunit	944–970 3′UTR	(Transcript) 5′GACCUAACAUUCGAUCUCAUUCAGUAUUA3′ (miRNA) 3′AGGUAGUAAUGGGCCGUCAUAAU5′	8mer
KDR	ENSG00000128052	Kinase insert domain receptor (VEGFR-2)	427–445 3′UTR	(Transcript) 5′CACUCUCACCCCGCAACCCAUCAGUAUUU3′ (miRNA) 3′AGGUAGUAAUGGGCCGUCAUAAU5′	7mer
MAP3K5	ENSG00000197442	Mitogen-Activated Protein Kinase Kinase Kinase 5	595–617 3′UTR	(Transcript) 5′AAAGGCGCUGCACUUUAAAUCCAGUAUUU3′ (miRNA) 3′AGUAAUGGGCCGUCAUAAU5′	7mer
PIK3CA	ENSG00000121879	Protein Phosphatase 2 Catalytic Subunit Alpha	702–708 3′UTR	(Transcript) 5’UUUUUUUCUUCUGGACAGUAUUU3′ (miRNA) 3’AGGUAGUAAUGGGCCGUCAUAAU5′	7mer-8m
PLCG1	ENSG00000124181	Phospholipase C Gamma 1	618–640 3′UTR	(Transcript) 5′AGGGGAUCAUGUUAAAAAUAGCAGUAUUA3′ (miRNA) 3′AGGUAGUAAUGGGCCGUCAUAAU5′	9mer
PPP2CA	ENSG00000113575	Protein Phosphatase 2 Catalytic Subunit Alpha	19–25 3′UTR	(Transcript) 5’AAUUUUAAACUUGUACAGUAUUG3′ (miRNA) 3’AGGUAGUAAUGGGCCGUCAUAAU5′	7mer-m8
RAP1B	ENSG00000127314	RAP1B, Member Of RAS Oncogene Family	235–256 3′UTR	(Transcript) 5′GGAAAACAGAGGCUACAUCCAGUAUUA3′ (miRNA) 3′AGGUAGUAAUGGGCCGUCAUAAU5′	8mer
RPS6KB1	ENSG00000108443	Ribosomal protein S6 kinase B1	291–298 3′UTR	(Transcript) 5’AUGACUCGAAACUGACAGUAUUA3′ (miRNA) 3’AGGUAGUAAUGGGCCGUCAUAAU5′	8mer
VEGFA	ENSG00000112715	Vascular endothelial growth factor A	1284–1303 3′UTR	(Transcript) 5′UUAAAGAGUAGGGUUUUUUUUCAGUAUUC3′ (miRNA) 3′GGUAGUAAUGGGCCGUCAUAAU5′	7mer
YWHAG	ENSG00000170027	Tyrosine 3-Monooxygenase/Tryptophan 5-Monooxygenase	564–571 3′UTR	(Transcript) 5’UCUCUGAUUAGUUGACAGUAUUA3′ (miRNA) 3’AGGUAGUAAUGGGCCGUCAUAAU5′	8mer

TargetScan Human, release 7.2 (www.targetscan.org, accessed on 3 June 2021) and Diana-TarBase v8, (http://carolina.imis.athena-innovation.gr/diana_tools/web/index.php?r=tarbasev8%2Findex, accessed on 3 June 2021) databases were used to predict the interaction sites between the transcripts and miR-200c-3p. 7mer-m8; sites complementary to miRNA nucleotide 2–8. Blue nucleotides are the predicted consequential pairing of target region transcript and miRNA.

## Data Availability

Not applicable.

## Supplementary Materials

The supporting information can be downloaded at: https://www.mdpi.com/article/10.3390/ijms232415659/s1. Reference [58] are cited in the supplementary materials.
