# Supplementary material for: Deciphering the Role of miR-200c-3p in Type 1 Diabetes (Subclinical Cardiovascular Disease) and Its Correlation with Inflammation and Vascular Health
Source: Int J Mol Sci. 2022 Dec 10;23(24):15659. doi: 10.3390/ijms232415659 (PMC9778946; doi:10.3390/ijms232415659)
Supplement: Supplementary file 1 [file ijms-23-15659-s001.zip › ijms-2054701-supplementary.pdf]

Supplementary Material

S1. Supplementary Figures

S1.1 Correlation between HbA1c and hsa-miR-200c-3p

Alongside the negative correlation, the residual plot produced from the HbA1c measurements gives a homoscedastic and unbiased representation of the results, with the majority of results lying within a 0.15 range of the modelled value produced by the linear regression; this reinforces the linear nature of the relationship between these variables (Figure S1 A).

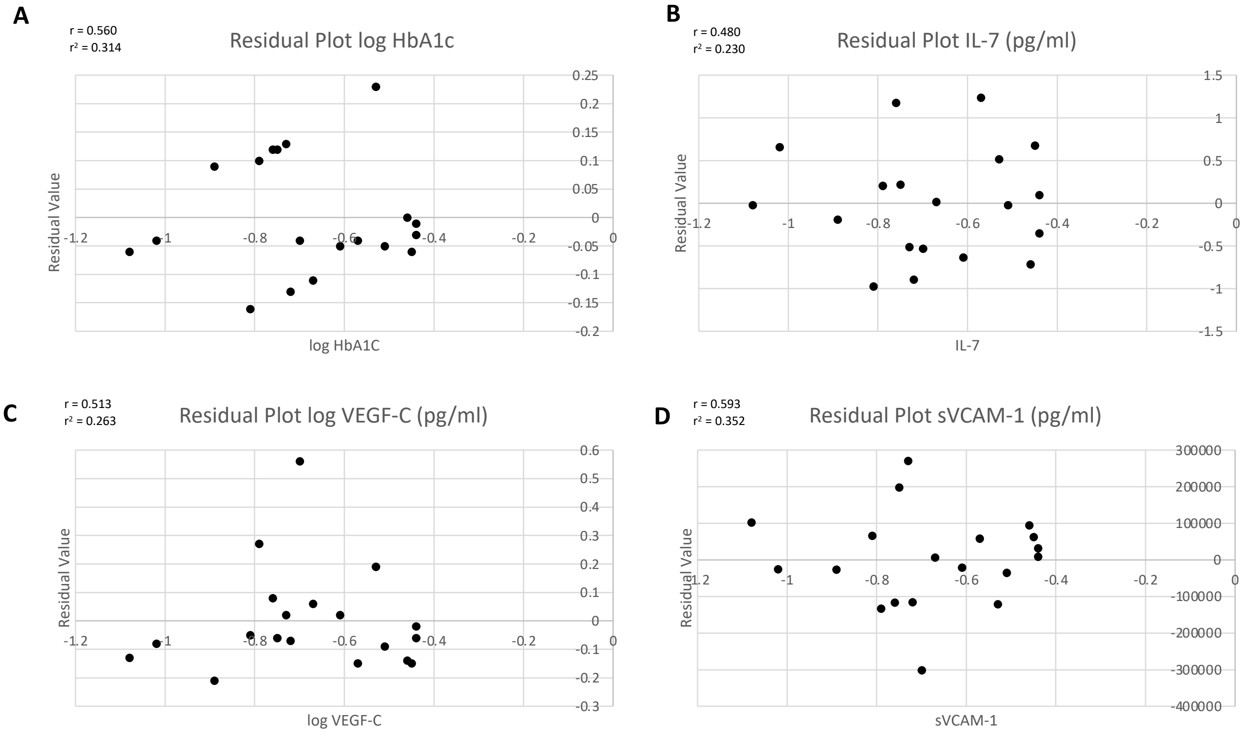

Figure S1: (**A**) Residual plot for correlation between miR-200c-3p in PBMCs and HbA_{1c}, (**B**) residual plot for correlation between miR-200c-3p in PMBC and IL7, (**C**) logVEGFC, (**D**) sVCAM-1. IL: interleukin; VEGFC: vascular endothelial growth factor C; sVCAM-1: circulating vascular cell adhesion molecule 1.

S1.2 Correlation between hsa-miR-200c-3p and cytokines

Residual plots constructed from IL-7 and sVCAM-1 measurements display centralized residual values in respect to miR-200c-3p expression, without bias or heteroscedasticity (Figure S1 B and D). This supports the nature of a linear relationship between the measurements of these cytokines and miR expression, where high expression of miR-200-3p is associated with lower levels of these cytokines. However, the residual plot constructed using log VEGF-C measurements displays a slight bias (due to an outlier), where the linear regression tends to underpredict the log VEGF-C measurement in relation to miR-200c-3p expression (Figure S1 C). Due to the small sample size, which consequently hinders the identification of possible erroneous outlying data points, it is difficult to fully understand the extent of this underprediction by linear regression, but still is homoscedastic in nature therefore the relationship between the variables can be presented linearly, but with caution.

S1.3 Correlation between miR-200c-3p and cEPCs

Residual values calculated and plotted for log CD45^{dim}CD34^{+}VEGFR-2^{+} and CD45^{dim}CD34^{+}CD133^{+} cell counts show no bias or patterns in relation to the expression of miR-200c-3p (Figure S2). This supports the concept of linearly modelling the significant positive correlation between miR-200c-3p expression and cell count in the subjects with natural variance and random error accounting for the range between residual values.

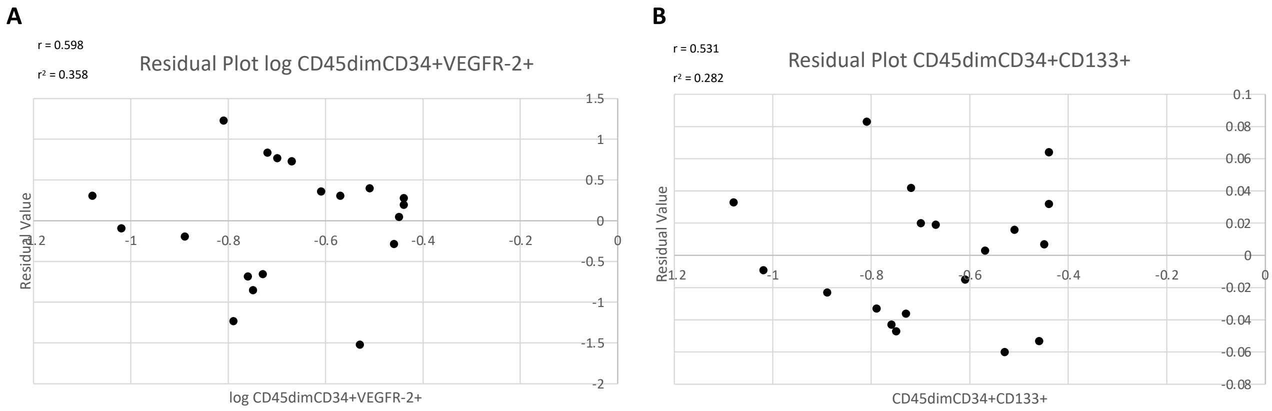

Figure S2: (**A**) Residual plot for correlation between miR-200c-3p in PMBC and logCD45^{dim}CD34^{+}VEGFR-2^{+}; (**B**) miR-200c-3p and CD45^{dim}CD34^{+}CD133^{+}. CD: cluster of differentiation; PAC: proangiogenic cells; VEGF: vascular endothelial growth factor.

S1.4 Correlation between miR-200c-3p and PACs

The significant positive linear relationship between PACs and miR-200c-3p expression shown in our data can be fortified by the homoscedastic residual plot, with even distribution of residual values above and below the predicted values calculated by the

regression; A linear model is therefore suitable and appropriate to describe the relationship between the variables.

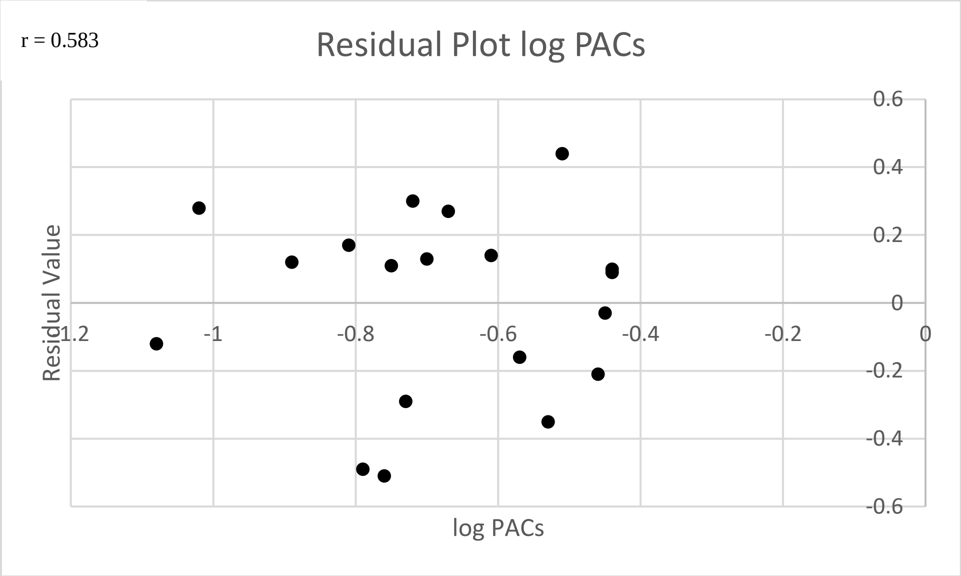

Figure S3: Residual plot for correlation between miR-200c-3p in PMBC and logPACs.

S1.5 Optimization of gating strategy for flow cytometric analysis of circulatory endothelial progenitor cells

BD FACSDiva™ software was used for flow cytometry data analysis. Forward and side scatter was plotted. Gate for leukocytes was drawn to exclude debris and beads. Subsequent analysis was done in the lymphocytic gate. Quantification of different cEPCs was performed by gating at the region containing lymphocyte sub-population of the cell. A further gate was set on CD45 vs. side scatter. This gate defined all $CD45^+$ positive events. The events in $CD45^+$ were then plotted on CD34 versus CD45. Gate was configured to define all events which were $CD34^+$ and $CD45^+$.

The lymphocytic gate was set on CD45 vs. side scatter. The gate was set to define all $CD45^{dim}$ positive events. $CD45^{dim}$ events were then plotted on CD34 versus CD45. The gate was set to define all events which were $CD34^+$ and $CD45^{dim}$ positive. Then we plotted all CD34 and $CD45^{dim}$ events on CD34 versus VEGFR2. Upper right quadrant was defined as $CD45^{dim}CD34^+VEGFR2^+$. Furthermore, all $CD34^+$ and $CD45^{dim}$ events were plotted as CD34 versus CD133. Upper right quadrant was defined as $CD45^{dim}CD34^+CD133^+$ (Figure S4).

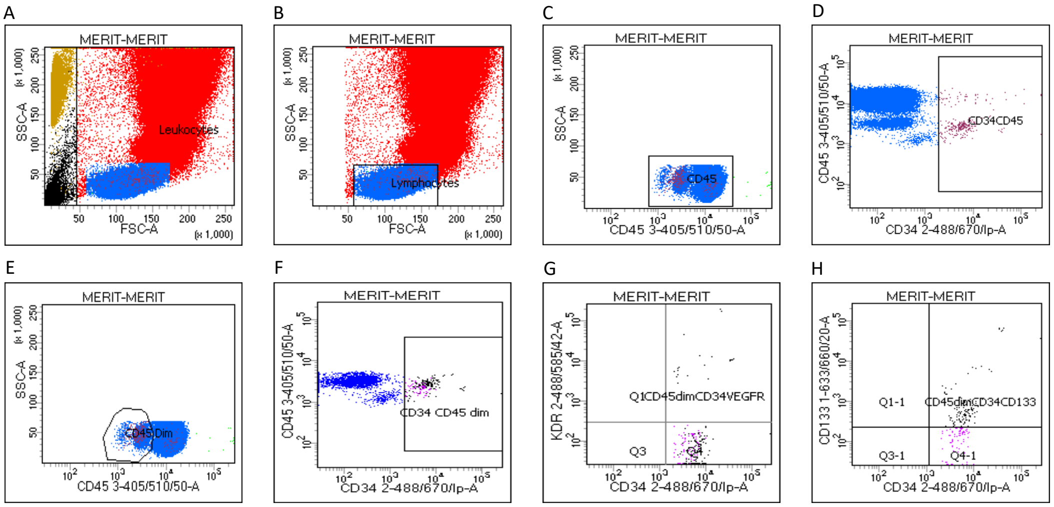

Figure S4: Gating strategy to identify cEPC. **A**. Debris (black) and Trucount beads (yellow) were excluded in side vs. forward-scatter graph; **B**. Gating selecting lymphocyte population; **C**. Gating selecting CD45 with all bright and dim population; **D**: Gating selecting CD45$^{+(\text{bright and dim})}$CD34^{+}; **E**. Gating selecting CD45^{dim}; **F**. Gating selecting CD45 ^{dim}CD34^{+}; **G**. Gating selecting CD45^{dim}CD34^{+}VEGFR2^{+}; **H**. Gating selecting CD45^{dim}CD34^{+}CD133^{+}.

S1.6 PAC staining with Ulex lectin and DiLDL

Staining EPCs were done according to the method discussed by Vasa *et al.*, 2001 [1]. Cells were incubated with DiLDL (1,19–dioctadecyl–3,3,39,39–tetramethylindocarbocyanine–labeled acetylated low-density lipoprotein) (2.4 ug/mL) for 1 hour at 37°C. 2% paraformaldehyde was used for 10minutes to fix the cells, and then lectin staining was performed by incubating the cells for 1 hour with fluorescein isothiocyanate (FITC)–labelled Ulex europaeus agglutinin I (lectin, ten ug/mL; Sigma). At least 15 randomly selected high-power fields were captured by an inverted microscope. Cells staining for both lectin and Di-LDL (Figure S5) were classed as PACs, and they were counted per high power fields.

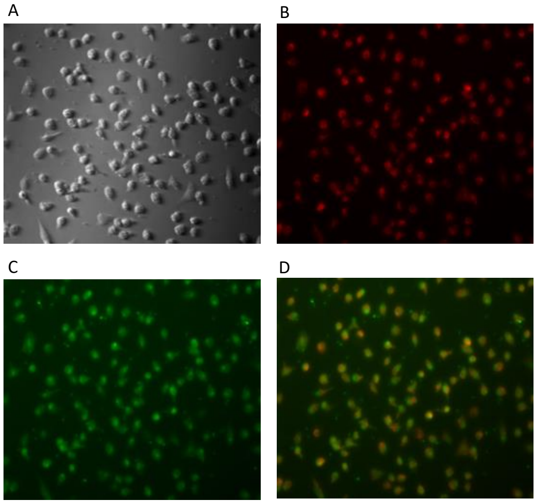

Figure S5: **A**. Pro-angiogenic cells (20×; Brightfield). **B**. Dil-LDL staining of EPCs (pro-angiogenic cells): Red. **C**. Ulex-Lectin staining of EPCs (pro-angiogenic cells): Green. **D**. Double staining of EPCs (superimposed image of EPCs labelled with dil-LDL and ulex-lectin).

S2. Supplementary Methods

S2.1 Quantitative real-time polymerase chain reaction for miRNA

miRNAs from PBMCs were normalized to the average of 11 assays (hsa-miR-30e-3p, Cat number: YP00204410; hsa-miR-365a-3p, Cat number: YP00204622; hsa-miR-374b-5p, Cat number: YP00204608; hsa-miR-26b-3p, Cat number: YP00204117; hsa-miR-576-5p, Cat number: YP00206064; hsa-miR-425-3p, Cat number: YP00204038; hsa-miR-454-5p, Cat number: YP00204279; hsa-miR-769-5p, Cat number: YP00204270; hsa-miR-200c-3p, Cat number: YP00204482; hsa-miR-660-5p, Cat number: YP00205911; hsa-miR-331-3p, Cat number: YP00206046; Qiagen) which was used for global mean normalization in qPCR.

References

1. Vasa, M.; Fichtlscherer, S.; Aicher, A.; Adler, K.; Urbich, C.; Martin, H.; Zeiher, A.M.; Dimmeler, S. Number and migratory activity of circulating endothelial progenitor cells inversely correlate with risk factors for coronary artery disease. *Circ. Res.* **2001,** *89*, e1–e7.
